# Supplementary material for: Performance of the GeneSoC Rapid PCR System in Detection of SARS-CoV-2 from Saliva Specimens
Source: Microbiol Spectr. 2023 Feb 13;11(2):e03259-22. doi: 10.1128/spectrum.03259-22 (PMC10101093; doi:10.1128/spectrum.03259-22)
Supplement: Supplemental file 1 — Supplemental material. Download spectrum.03259-22-s0001.pdf, PDF file, 0.04 MB [file spectrum.03259-22-s0001.pdf]

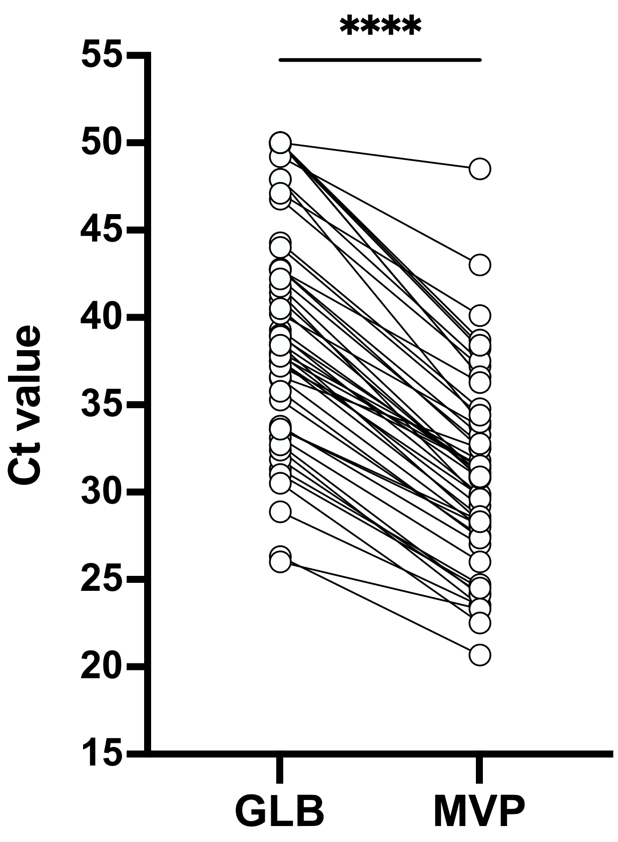
****
55
50
45
40
35
30
25
20
15
Ct value
GLB
MVP

Supplemental Figure 1. The cycle threshold (Ct) values determined by GeneSoC are shown, with lines connecting the results for the same sample tested in each. Five samples with negative results by GLB are expressed as Ct = 50. Results were compared with the use of a Paired t test. ****, $P < 0.0001$. GLB, GeneSoC Lysis Buffer kit; MVP, MagMAX Viral Pathogen kit.
